# Supplementary material for: Sar1 Affects the Localization of Perilipin 2 to Lipid Droplets
Source: Int J Mol Sci. 2022 Jun 7;23(12):6366. doi: 10.3390/ijms23126366 (PMC9223735; doi:10.3390/ijms23126366)
Supplement: Supplementary file 1 [file ijms-23-06366-s001.zip › ijms-1719621-supplementary.pdf]

Table S1. Sequences for shRNA.

shRNA	Sequences (5′-3′)
Human Sar1a	sense: GCAATTAATGGGATTGTCT
Human Sar1b	sense: GGACATGTTCAAGCTCGAA

Table S2. Sequences of qPCR primers.

qPCR primers	Sequences (5′-3′)
Human *GAPDH* (forward)	CCTGTTCGACAGTCAGCCG
Human *GAPDH* (reverse)	CGACCAAATCCGTTGACTCC
Human *PLIN2* (forward)	TCAGCTCCATTCTACTGTTCACC
Human *PLIN2* (reverse)	CCTGAATTTTCTGATTGGCACT

Table S3. Antibodies for immunoblotting and immunofluorescence.

Antibody	Source	Dilution	Company (#Catalogue)
PLIN2	rabbit	1:1000 (IB)	Proteintech (15294-1-AP)
		1:500 (IF)	
PLIN3	rabbit	1:2000 (IB)	Proteintech (10694-1-AP)
Calnexin	mouse	1:2000 (IB)	Sigma-Aldrich (C4731)
EEA1	mouse	1:1000 (IB)	BD Biosciences (610456)
GM130	mouse	1:500 (IB)	BD Biosciences (610822)
Lamp-1	mouse	1:3000 (IB)	BD Biosciences (611042)
Bcl-2	mouse	1:500 (IB)	BD Biosciences (610538)
Sec22b	rabbit	1:2000 (IB)	Synaptic Systems (186003)
GAPDH	rabbit	1:5000 (IB)	Sigma-Aldrich (G9545)
GFP	rabbit	1:2000 (IB)	MBL (598)
KDEL	mouse	1:1000 (IB)	ENZO (ENZ-ABS679)
Actin	mouse	1:1000 (IB)	Santa cruz (sc47778)
Nucleoporin	mouse	1:1000 (IB)	BD Biosciences (610497)
HRP-rabbit	donkey	1:5000 (IB)	GE healthcare (NA934)
HRP-mouse	sheep	1:2000 (IB)	GE healthcare (NA931)

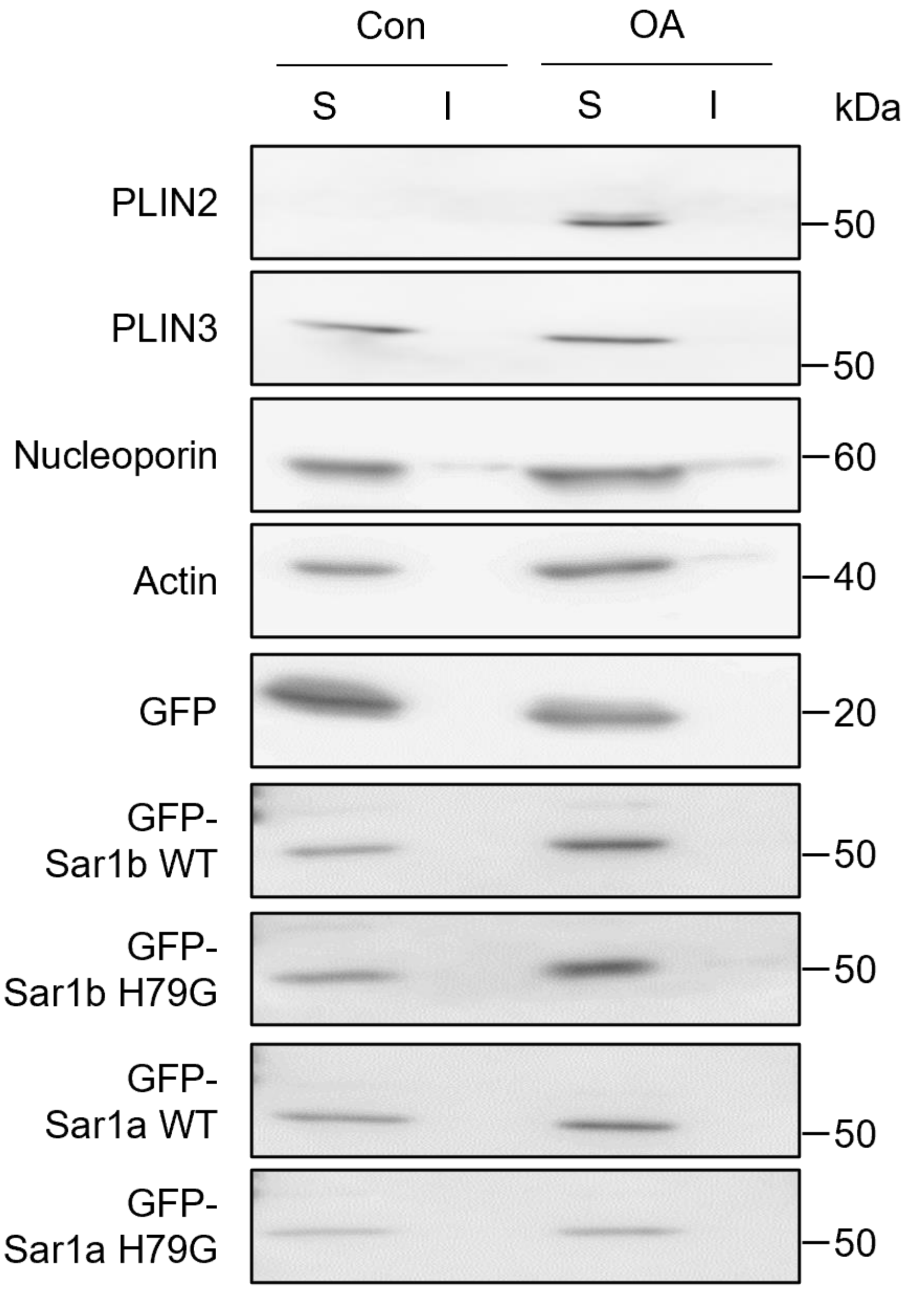

Figure S1. Recovery of cell proteins in soluble fraction during preparation of cell lysate. HuH7 cells transiently expressing GFP, GFP-Sar1b WT, GFP-Sar1b H79G, GFP-Sar1a WT, or GFP-Sar1a H79G were treated with (OA) or without (Con) 0.5 mM oleic acid for 24 h. Cells were treated with lysis buffer and sonicated for 10 sec on ice, and centrifugated at 15000 rpm at 4℃ for 10 min. The supernatant after the centrifugation was used as soluble fraction (S), and the palette (I) was lysed in SDS buffer (1% SDS, 20 mM Tris (pH7.5), 2 mM EDTA, 2 mM EGTA and proteinase inhibitor cocktail) was used as insoluble fraction (I). Same amount of each fraction was analyzed by western blotting using the indicated antibodies.

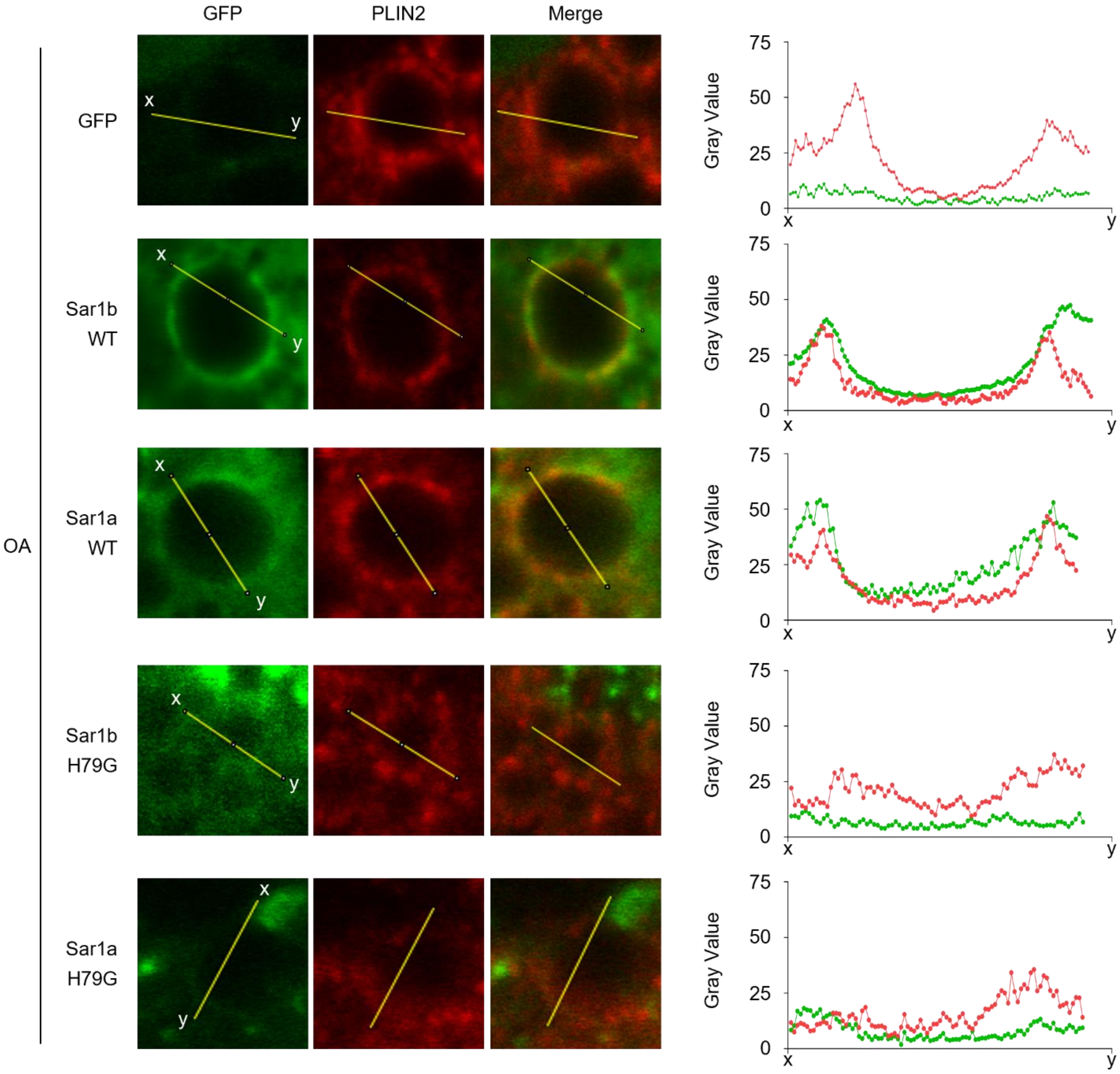

Figure S2. Line profile analysis of lipid droplets in the cell. Higher magnification of the results in Fig4 and Fig5 were shown (left). The results of line profile analysis of the fluorescence images obtained in left panel were shown as gray scale (right).

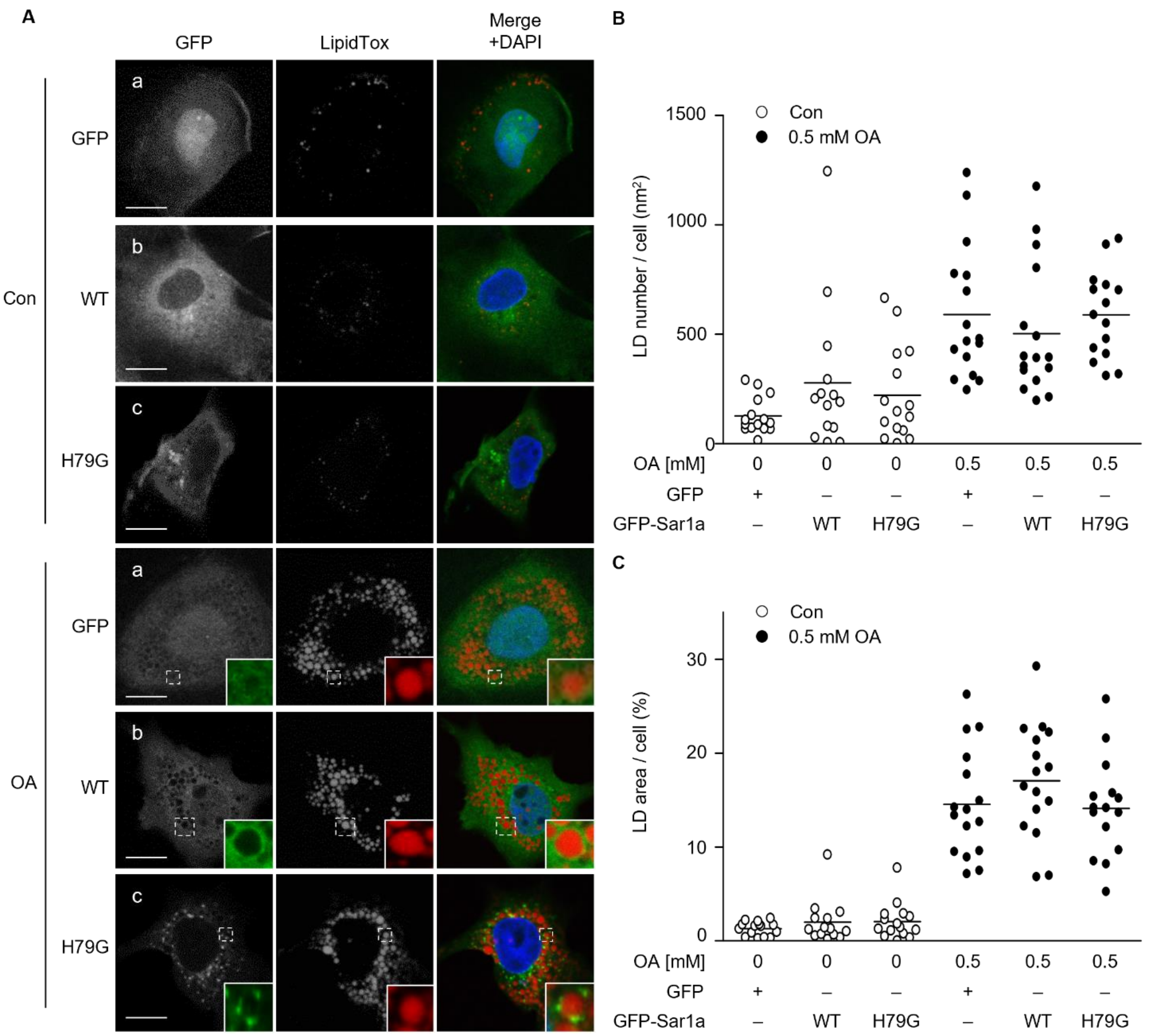

Figure S3. Effect of Sar1a on lipid droplet formation. (A) HuH7 cells transiently expressing GFP (a), GFP-Sar1a WT (b), or GFP-Sar1a H79G (c) were treated with (OA) or without (Con) 0.5 mM oleic acid for 24 h. The cells were stained with LipidTox and DAPI. The boxed areas are shown in higher magnification in the insets. Bar, 10 μm. The data are representative from experiments repeated more than three times. (B) The number of LDs per cell area in (A) are shown. Results are shown as a scattered dot plot. (C) The ratio of LD area to total cell area in (A) are shown. Results are shown as a scattered dot plot.

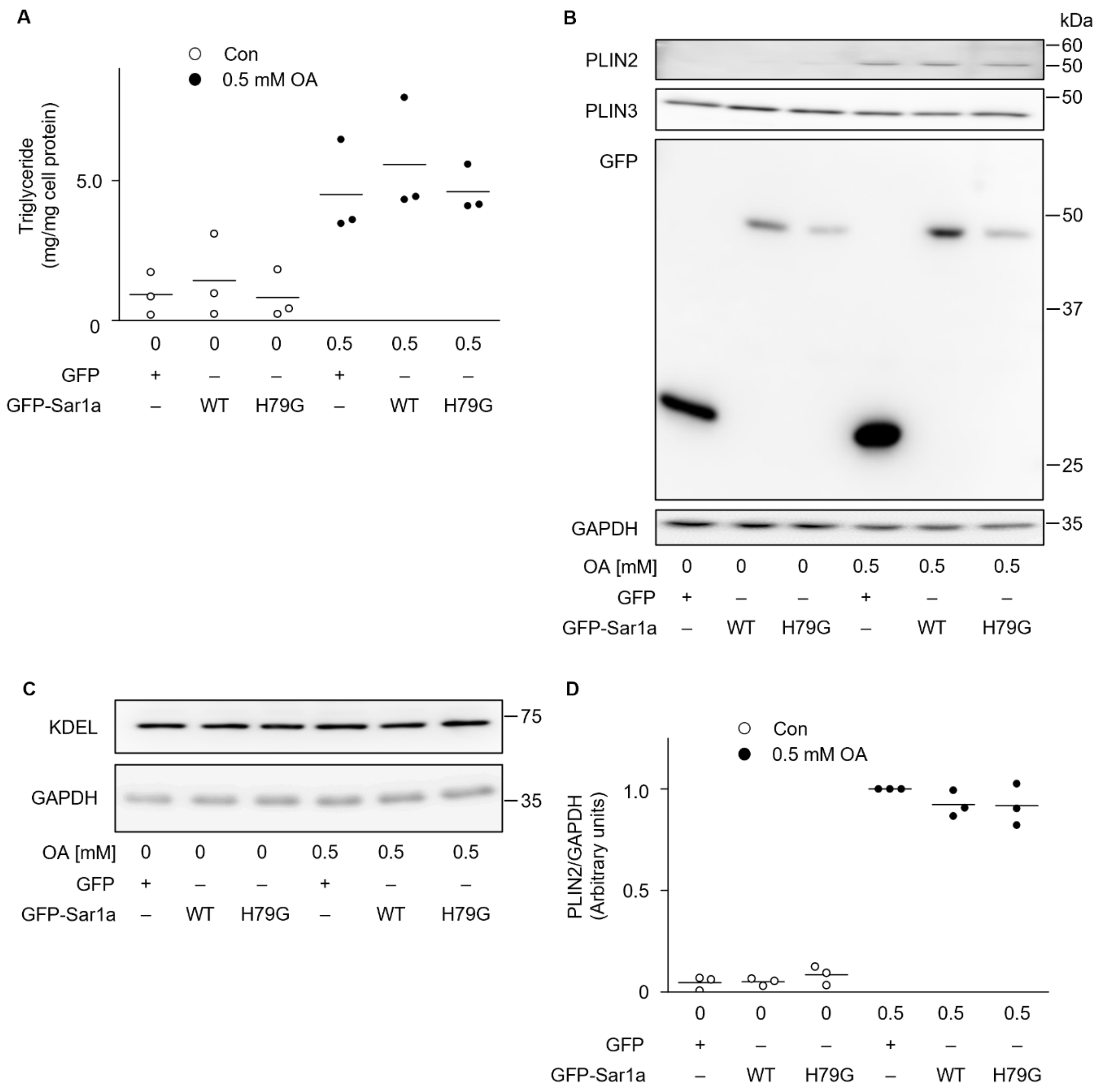

Figure S4. Effect of Sar1a on PLIN2 expression in the cells. HuH7 cells transiently expressing GFP, GFP-Sar1a WT, or GFP-Sar1a H79G were treated with (OA) or without (Con) 0.5 mM oleic acid for 24 h. (A) The intracellular TG levels were quantified. Results are shown as a scattered dot plot. (B, C) Cell lysates were analyzed by western blotting using the antibodies to the indicated proteins. Data are representative from experiments repeated more than three times. (D) The ratio of PLIN2 protein level relative to GAPDH is expressed as arbitrary units. PLIN2 level in OA treated cells expressing GFP was set to 1.0. Results are shown as a scattered dot plot.
